# Optimization of a 3D Dynamic Culturing System for *In Vitro* Modeling of Frontotemporal Neurodegeneration-Relevant Pathologic Features

**DOI:** 10.3389/fnagi.2016.00146

**Published:** 2016-06-22

**Authors:** Marta Tunesi, Federica Fusco, Fabio Fiordaliso, Alessandro Corbelli, Gloria Biella, Manuela T. Raimondi

**Affiliations:** ^1^Department of Chemistry, Materials and Chemical Engineering “Giulio Natta”, Politecnico di MilanoMilan, Italy; ^2^Unità di Ricerca Consorzio INSTM, Politecnico di MilanoMilan, Italy; ^3^Department of Neuroscience, IRCCS-Istituto di Ricerche Farmacologiche “Mario Negri”Milan, Italy; ^4^Unit of Bio-imaging, Department of Cardiovascular Research, IRCCS-Istituto di Ricerche Farmacologiche “Mario Negri”Milan, Italy

**Keywords:** neurodegeneration, exosomes, progranulin, 3D cell culture, bioreactor, microfluidic, scaffold, cell modeling

## Abstract

Frontotemporal lobar degeneration (FTLD) is a severe neurodegenerative disorder that is diagnosed with increasing frequency in clinical setting. Currently, no therapy is available and in addition the molecular basis of the disease are far from being elucidated. Consequently, it is of pivotal importance to develop reliable and cost-effective *in vitro* models for basic research purposes and drug screening. To this respect, recent results in the field of Alzheimer’s disease have suggested that a tridimensional (3D) environment is an added value to better model key pathologic features of the disease. Here, we have tried to add complexity to the 3D cell culturing concept by using a microfluidic bioreactor, where cells are cultured under a continuous flow of medium, thus mimicking the interstitial fluid movement that actually perfuses the body tissues, including the brain. We have implemented this model using a neuronal-like cell line (SH-SY5Y), a widely exploited cell model for neurodegenerative disorders that shows some basic features relevant for FTLD modeling, such as the release of the FTLD-related protein progranulin (PRGN) in specific vesicles (exosomes). We have efficiently seeded the cells on 3D scaffolds, optimized a disease-relevant oxidative stress experiment (by targeting mitochondrial function that is one of the possible FTLD-involved pathological mechanisms) and evaluated cell metabolic activity in dynamic culture in comparison to static conditions, finding that SH-SY5Y cells cultured in 3D scaffold are susceptible to the oxidative damage triggered by a mitochondrial-targeting toxin (6-OHDA) and that the same cells cultured in dynamic conditions kept their basic capacity to secrete PRGN in exosomes once recovered from the bioreactor and plated in standard 2D conditions. We think that a further improvement of our microfluidic system may help in providing a full device where assessing basic FTLD-related features (including PRGN dynamic secretion) that may be useful for monitoring disease progression over time or evaluating therapeutic interventions.

## Introduction

Neurodegenerative diseases share common pathological features, such as abnormal protein aggregation, mitochondrial dysfunction, and disease-specific neuronal degeneration. Frontotemporal lobar degeneration (FTLD) is a severe neurodegenerative disorder that is diagnosed with increasing frequency in clinical setting. It is related to the progressive and selective degeneration of the frontal lobes, temporal lobes or both, with relative preservation of the posterior cortical regions. Its clinical symptoms span from behavioral/dysexecutive syndrome, language disorders and motor disorders, but the molecular basis are far from being elucidated and involve several molecular players including the inflammation-related protein progranulin (PRGN; Pressman and Miller, 2014; Galimberti et al., 2015; Irwin et al., 2015).

FTLD therapies aiming at reducing the accumulation of tau protein and increasing PRGN levels have been explored (Boxer et al., 2013).

Primary neurons or neuronal-like SH-SY5Y cells are extensively used as *in vitro* models for neurodegenerative disorders. Glass or plastic two-dimensional (2D) surfaces are important tools to investigate key features of homogeneous cell populations in an isolated system, but they show some limitations to assess the relationship between anatomical and functional connectivity (Bosi et al., 2015). Conventional 2D culture systems fail to provide topographical cues, enable cell differentiation into specific phenotypes and fully reproduce *in vivo* cell behavior (Lai et al., 2012); furthermore dissociated neurons cannot be cultured for several weeks. Organotypic cell cultures allow for a more realistic mimic of the *in vivo* situation, since they permit the simultaneous culturing of cells from functionally related brain regions while preserving natural cell-to-cell physical contacts (Benam et al., 2015). The complex physiological three-dimensional (3D) architecture is partially maintained, but pathways are largely disconnected because of harvesting injury. Again, the observation time window may be limited to a few weeks (Cavaliere et al., 2010; Humpel, 2015; Geuna et al., 2016). To provide structural and biochemical cues together with a suitable stiffness and a better control of the cellular context, 3D matrices such as hydrogels, spheroid-based systems or porous materials have been exploited as 3D microenvironments (Yamada and Cukierman, 2007; Zhang et al., 2014). Their main limitation is related to oxygen and nutrient diffusion. In isolated tissues, it is limited to 100–200 μm and its availability may influence cell growth and differentiation (Colom et al., 2014; Liu et al., 2015). Dynamic culture systems can overcome limited mass transport, thus contributing to the extension of the culturing time. In these systems, cells are cultured under a continuous flow of growing medium, thus mimicking the interstitial fluid movement that actually perfuses the body tissues, including the brain. They may also provide fluid mechanical loading acting as a physiologically relevant stimulus for cell metabolic activity, if suitable mathematical and computational models are used to achieve the proper bioreactor design (Sacco et al., 2011). In addition, dynamic conditions may better reproduce pathology-relevant features, such as protein seeding and spreading across cells, that is relevant also for FTLD (Jucker and Walker, 2011). However, together with the accumulation of abnormal protein aggregates, other mechanisms such as oxidative stress are involved in the degenerative process, as reported in FTLD linked to mutations in tau protein (Martínez et al., 2008). Martínez et al. (2008) have evidenced the presence of differences in the extent of oxidative damage among the types of FTLD; in particular, in FTLD-tau patients they have observed enhanced lipoxidative damage in the frontal cortex. Finally, they have suggested that astrocytes are targets of lipoxidative damage, glial fibrillary acidic protein is modified by oxidation and astrocytes play a key role in oxidative stress response.

Microfluidic technology shows several advantages for 3D cell culture, such as the possibility to model complex dynamic microenvironments mimicking the *in vivo* situation in a controlled and tunable way. In fact, this technique allows to achieve spatial and temporal control over flows and velocity gradients in micrometer-sized channels, contributing to extend the observation time window (Li et al., 2012; van Duinen et al., 2015). As an example, Moreno et al. (2015) have cultured human induced pluripotent stem cell-derived neuroepithelial stem cells differentiated into dopaminergic neurons for 30 days in arrays of microfluidic bioreactors in a microtiter plate format.

In this work, we have optimized a miniaturized, microfluidic, 3D scaffold-based perfusion bioreactor as a simple, reproducible and reliable platform for *in vitro* modeling of basic research features of FTLD.

## Materials and Methods

### The Miniaturized, Microfluidic and Optically Accessible Bioreactor

**Figure 1:**
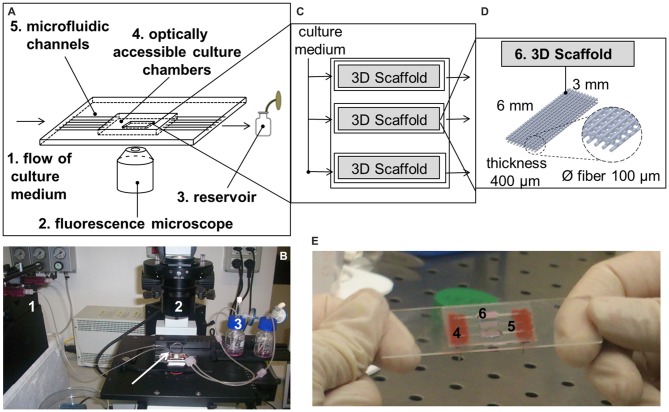
**The miniaturized, microfluidic and optically accessible bioreactor. (A)** Sketch of the main components of the 4th dimensional (4D) bioreactor; **(B)** The 4D bioreactor is placed on a standard inverted fluorescence microscope and connected to a syringe pump. The arrow indicates a short, gas-permeable tubing that may be used to connect one chamber to another; **(C)** Sketch of the culture system hosting the tridimensional (3D) cell-seeded scaffolds; **(D)** Sketch of the 3D polystyrene scaffolds; **(E)** Representation of the culture chambers after the positioning of the 3D cell-seeded scaffolds.

The miniaturized, microfluidic bioreactor for interstitial perfusion of 3D cell-seeded scaffolds (Figure 1) was preliminarily described in a previous study (Laganà and Raimondi, 2012). Thanks to the optical transparency and low thickness of the components, it is optically accessible and may be exploited as a 4D bioreactor for prolonged culture (the 4th dimension is represented by time). It means that it may be used to assess the effect of several parameters on engineered tissue growth with time by viable staining and standard fluorescence microscopy. This dynamic culture system is composed of three independent chambers, each hosting one independent scaffold. The chambers are perfused by three independent microfluidic channels directly machined in a standard microscope glass slide. Culture medium is pumped with a flow rate of 5.0 × 10^−3^ ml/min by a multi-channel programmable syringe pump (PHDULTRA, Harvard Apparatus, Holliston, MA, USA) in a gas permeable tubing system (0.03″ ID × 0.065″ OD, Silastic^®^ Tubing, Cole-Parmer, Vernon Hills, IL, USA) and collected in reservoirs after a single passage. The scaffolds (of dimensions 6 × 3 × 0.4 mm, 3D Biotek, Hillsborough, NJ, USA) are made up of polystyrene. They are obtained by fuse deposition modeling and they are composed of four layers of fibers (100 μm diameter, with a pore size of 300 μm) shifted of 150 μm with respect to the adjacent. After placing the scaffolds, the culture chambers are sealed by a microscope cover-glass coated with poly(dimethylsiloxane) (PDMS) and then locked by magnetic holders.

### Cell Culture and Experimental Design

SH-SY5Y human neuroblastoma cells (ATCC^®^ code CRL-2266^TM^) are a widely used model for neurodegenerative disorders. They were cultured at 37°C and 5% CO_2_ in high-glucose Dulbecco’s modified Eagle’s medium supplemented with 10% (v/v) fetal bovine serum, 2 mM L-glutamine, 100 U/ml penicillin and 100 μg/ml streptomycin sulfate (EuroClone, Pero, Italy) and split twice a week.

**Figure 2:**
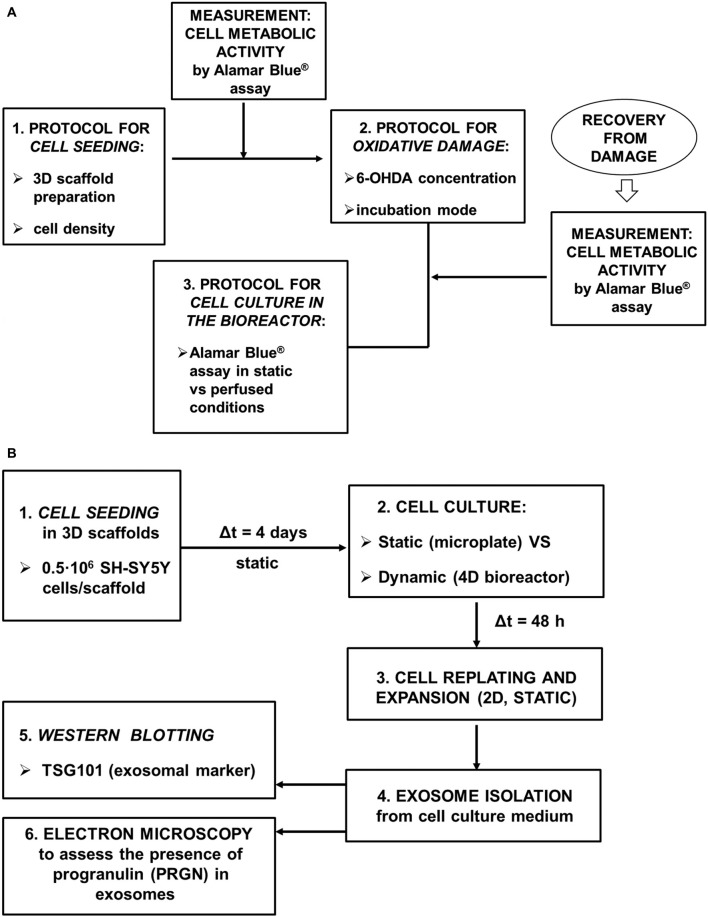
**Schematic picture of the experimental design. (A)** The following experimental procedures were optimized: seeding of SH-SY5Y neuronal-like cells in 3D scaffolds, oxidative damage in 3D cell-seeded scaffolds by a mitochondrial-targeted toxin (6-OHDA), evaluation of cell metabolic activity while in the 4D bioreactor; **(B)** Exosome production and characterization.

To adapt our 4D microfluidic bioreactor for use in the assessment of potential therapeutic strategies against neurodegeneration, we have focused on the optimization of the following experimental protocols (Figure 2A):

1. Cell seeding in 3D scaffolds;
2. Oxidative damage in 3D cell-seeded scaffolds;
3. Evaluation of cell metabolic activity while in dynamic culture.

Finally, we have focused on the demonstration that SH-SY5Y cells retain their basic capacity to secrete PRGN in exosomes once recovered from the 4D bioreactor (Figure 2B).

### Seeding of Neuronal-like Cells

To increase the seeding efficiency of neuronal-like cells (that is the number of cells adhering to the 3D scaffolds), the seeding protocol was optimized starting from a procedure previously developed for MG-63 osteoblast-like cells (Laganà and Raimondi, 2012). In sterile conditions, scaffolds were placed (preferentially in the vertices) in an ultra-low attachment 24-well microplate (Corning^®^ Costar^®^, Corning, NY, USA) and incubated overnight with 400 μl culture medium. The following day, medium was replaced with cell suspension (two seeding densities were tested: 0.5 and 1·10^6^ SH-SY5Y cells/400 μl). To achieve full 3D seeding the plate was moved to the incubator and shaken for 3 h by a 3D multifunction rotator (PS-M3D, Grant Instruments, VWR, Shepreth, UK) with a protocol alternating mixing, bending and vibration. To enhance the efficacy of the dynamic mixing and expose all the samples to the maximum bending, the microplate was manually rotated clockwise every 15–20 min. The following day the 3D cell-seeded scaffolds were moved to empty wells and incubated with 400 μl medium.

To estimate the number of cells seeded in 3D scaffolds, the samples were incubated with 200 μl sterile distilled water (EuroClone) and subjected to repeated cycles of freezing at −80°C and thawing at 37°C (30 min each). To improve cell detachment, during thawing the microplate was placed on an orbital shaker (Rotamax 120, Heidolph, Schwabach, Germany). Two microlitres lysate was pipetted on a NanoQuant Plate^TM^ and DNA concentration was estimated by measuring the absorbance (Abs) at 260 nm (Infinite M200 PRO, Tecan, Männedorf, Switzerland). The blank was automatically subtracted and a ratio of ~1.8 was accepted for Abs_260_/Abs_280_, indicating that DNA was of good quality (Zhou et al., 1996). Since every human diploid cell contains about 6.4 pg DNA (Doležel et al., 2003), the number of neuronal-like cells was calculated as:

$$\text{cell ​​number}\;=\;\frac{\text{DNA concentration}\left[\frac{\text{ng}}{\text{μl}}\right]\cdot \text{volume}\left[\text{μl}\right]}{6.4\left[\text{pg}\right]}\cdot 1000$$

The experiment was run in triplicate and the results were reported as mean ± standard deviation (SD). As a reference, the experiment was also performed on 0.5·10^6^ SH-SY5Y cells plated in a standard 24-well microplate.

### Oxidative Damage

SH-SY5Y cells were exposed to 6-OHDA (Sigma-Aldrich, St. Louis, MO, USA), a toxin applied to produce oxidative and mitochondrial damage, a feature relevant also for FTLD models. For example, Bannwarth et al. (2014) identified a FTLD-amyotrophic lateral sclerosis phenotype in a mitochondrial disease. To obtain an effective, reproducible and long-lasting neuronal cell damage, two protocols were compared:

1. One day after seeding (1·10^6^ SH-SY5Y cells), the samples were incubated overnight with 6-OHDA (250 or 500 μM) in static conditions;
2. Four days after seeding (0.5·10^6^ SH-SY5Y cells), the samples were incubated overnight with 6-OHDA (125 or 250 μM) while shaken on a 3D multifunction rotator with a protocol alternating mixing and bending.

The 6-OHDA-induced neurotoxicity and its toxic effect over time were evaluated in terms of reduction of cell metabolic activity by Alamar Blue^®^ colorimetric assay (resazurin sodium salt, Sigma-Aldrich). Resazurin is oxidized by mitochondria and this process results in a color shift from purple/blue to pink. The amount of color absorption is related to the amount of metabolically active cells. Culture medium was replaced with 400 μl working solution (90% (v/v) culture medium, 10% (v/v) 0.2 mg/ml resazurin sodium salt in phosphate buffered saline solution, PBS). After 3 h at 37°C and 5% CO_2,_ 100 μl supernatant was transferred (in triplicate) in a 96-well microplate (Corning^®^ Costar^®^). Cell metabolic activity was estimated by measuring Abs_570_ (reference wavelength = 600 nm) or the fluorescence at 590 nm (after excitation at 560 nm). The experiment was run in triplicate and the results were reported as mean ± SD after subtracting the blank.

As an internal control to confirm the toxicity of 6-OHDA, 0.5·10^6^ SH-SY5Y cells were seeded in a 24-well microplate and incubated with 6-OHDA (250 or 500 μM) in static conditions the following day.

### Metabolic Activity in Dynamic Culture

To exploit the miniaturized bioreactor as a 4D culture system for quantitative evaluation with time, we identified the conditions in which comparable results are provided by Alamar Blue^®^ assay performed in static or dynamic conditions. 3D scaffolds were seeded with 0.5·10^6^ SH-SY5Y cells. After verifying that cell metabolic activity was comparable among the samples (and thus that seeding was a reproducible process), the cell-seeded scaffolds were cultured in static conditions in an ultra-low attachment 24-well microplate for 2 days. Then they were divided into two groups: the static (i.e., kept in the ultra-low attachment microplate) and the dynamic (i.e., assembled in the 4D bioreactor, where every culture chamber was connected to a 1 ml syringe and a reservoir). Cell metabolic activity was evaluated by Alamar Blue^®^ assay. Static constructs were incubated with 400 μl working solution, while dynamic constructs were perfused with working solution at a flow rate of 5.0 × 10^−3^ ml/min. After 3 h, the fluorescence of supernatants from static (400 μl each) and dynamic (900 μl each) samples was measured. Finally, the supernatants from static samples were diluted with PBS to 900 μl/sample and their fluorescence was measured again. The experiment was run in triplicate and the results were reported as mean ± SD after subtracting the blank.

### Cell Recovery From the Bioreactor and Replating

To demonstrate that SH-SY5Y cells retain their basic capacity to secrete PRGN in exosomes once recovered from the 4D bioreactor and plated in standard 2D conditions, the neuronal-like cells were seeded in 3D scaffolds (0.5·10^6^ SH-SY5Y cells/scaffold). Four days later they were assembled in the 4D bioreactor, where every culture chamber was connected to a 20 ml syringe and a reservoir. The constructs were perfused at a flow rate of 5.0 × 10^−3^ ml/min for 48 h (dynamic condition), then cells were detached, counted, plated in a 12-well microplate and expanded in a T25 cm^2^ flask and then in T75 cm^2^ flasks for exosome isolation.

As a control, SH-SY5Y cells were also seeded in 3D scaffolds and kept in an ultra-low attachment 24-well microplate (static condition). The experiment was run in triplicate.

### Isolation of Exosomes from Cell Culture Medium

To test SH-SY5Y cell ability to secrete the frontotemporal dementia-related protein PRGN, the exosomal fraction was collected after the cells were kept for 48 h in serum-free medium. Serum-free media (40 ml/condition, from 6 T75 cm^2^ flasks/condition) were collected and centrifuged at 300 g for 10 min (4°C) to eliminate cellular debris. The supernatants were centrifuged at 2000 g for 10 min (4°C), at 10,000 g for 30 min (4°C) and finally at 100,000 g for 70 min (4°C). The supernatants were removed and pellets (exosomes) were suspended in 20 μl RIPA lysis buffer (Thermo Fisher Scientific, Waltham, MA, USA) for further analysis.

### Western Blotting

To assess the presence of exosomes, recovered ultracentrifuged pellets and cell lysates containing 20 μg total proteins (as assessed by BCA Protein Assay Kit, Pierce^TM^, Thermo Fisher Scientific) were subjected to 8% SDS-PAGE electrophoresis and transferred to a nitrocellulose membrane (BioRad Laboratories, Hercules, CA, USA). The membrane was incubated with the exosome marker TSG101 monoclonal primary antibody (1:500, Abcam, Cambridge, UK) for 2 h, then with a horseradish peroxidase-conjugated secondary antibody (1:2000, Jackson ImmunoResearch, West Grove, PA, USA) and finally ECL-detected (Millipore, Billerica, MA, USA). To semi-quantitatively compare exosome production from dynamic and static samples, the ratio between the signal from cell lysate and the corresponding pellet recovered from ultracentrifugation was quantified in the resulting impressed film with a free image analyzer (ImageJ^1^).

### Transmission Electron Microscopy

To characterize the exosomal presence of PRGN, immuno-electron microscopy was performed on isolated exosomes from neuronal-like cells recovered from the 4D bioreactor. A 5 μl drop of purified exosomes in PBS was placed on a 100 mesh formvar/carbon coated copper grid (EMS, Hatfield, PA, USA), dried at room temperature for 30 min and fixed with with 4% paraformaldehyde and 0.2% glutaraldehyde in 0.12 M phosphate buffer (pH 7.4) for 30 min. Exosomes were then incubated overnight at 4°C with a rabbit anti-human PRGN antibody (1:100, Invitrogen, Carlsbad, CA, USA. The specificity of this antibody is detailed in “Supplementary Figure 1”), followed by a donkey anti-rabbit antibody conjugated to a 12 nm colloidal gold (1:75, Jackson ImmunoResearch) in block solution for 45 min at 37°C. After post-fixation with 2% glutaraldehyde, grids were finally counterstained with uranyl acetate, embedded in LR White and observed with an Energy Filter Transmission Electron Microscope (EFTEM, ZEISS LIBRA^®^ 120, Oberkochen, Germany) equipped with YAG scintillator slow scan CCD camera.

### Statistical Analysis

Statistical analysis was performed with GraphPad Prism^®^ software (GraphPad Software, Inc.). Two-way analysis of variance (ANOVA) for repeated measurements followed by Tukey’s multiple comparisons test was used for comparisons among the groups and time frames, while one-way ANOVA followed by Tukey’s multiple comparisons test was used for comparisons among the groups. The significance level was set at *p*-value < 0.05.

## Results

### Seeding of SH-SY5Y Neuronal-Like Cells on 3D Scaffold

**Figure 3:**
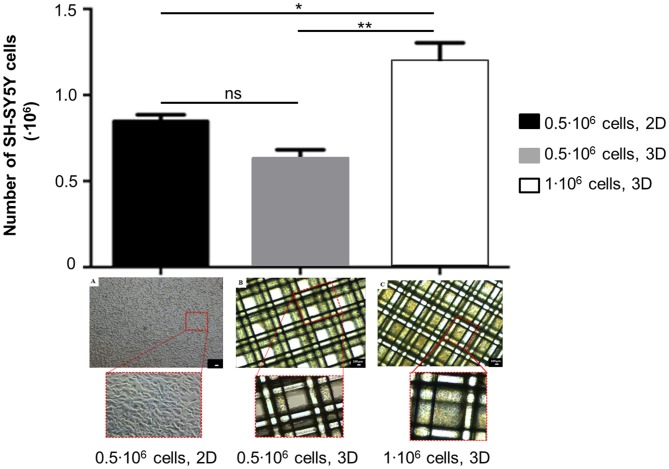
**Assessment of cell number and morphology in 3D scaffolds seeded with 0.5·10^6^ or 1·10^6^ SH-SY5Y cells (mean ± SD, *n* = 3).** The seeding of 0.5·10^6^ cells in 2D was considered as a reference. Statistical analysis was performed with one-way ANOVA followed by Tukey’s multiple comparisons test. Ns, *p*-value > 0.05; *p-value < 0.05; **p-value < 0.01. Optical micrographs of: **(A)** 0.5·10^6^ cells/well, 2D; **(B)** 0.5·10^6^ cells/scaffold; **(C)** 1·10^6^ cells/scaffold. Magnification = 4×, scale bar = 100 μm.

To evaluate the optimal seeding conditions in 3D scaffolds, DNA content was measured about 24 h after seeding, then the number of SH-SY5Y cells in 3D scaffolds was estimated as reported in Methods. As shown in Figure 3, statistical analysis has indicated that after seeding 0.5·10^6^ SH-SY5Y cells, the number of cells in 3D scaffolds was comparable to the corresponding seeding in 2D condition (ns, *p*-value > 0.05). As expected, when 1·10^6^ SH-SY5Y cells were seeded, a greater number of cells was obtained in 3D scaffolds (**p-value < 0.01) also with respect to the 2D condition (*p-value < 0.05), thus confirming that the procedure did not lead to major cell loss. Morphological analysis has revealed pore occlusion in the scaffolds seeded with 1·10^6^ cells because of cell clusters; while the scaffolds seeded with 0.5·10^6^ cells have shown a more homogeneous cell distribution on the fibers.

### Oxidative Damage on 3D Scaffolds

**Figure 4:**
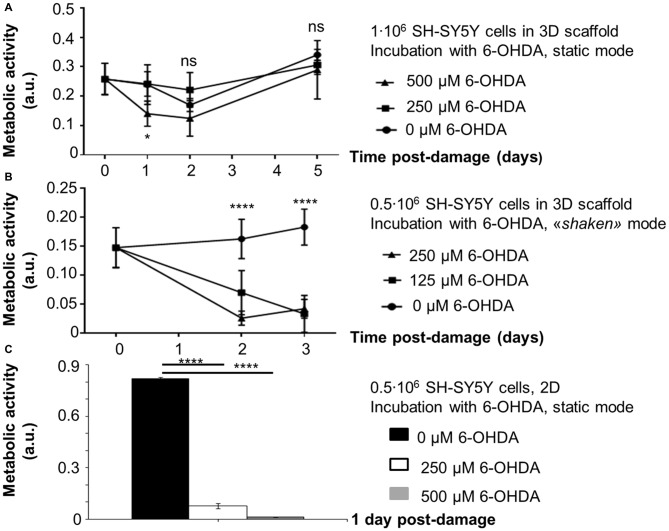
**Evaluation of 6-OHDA challenge on SH-SY5Y cells (absorbance values, mean ± SD, *n* ≥ 3).** Metabolic activity as a function of time post damage for: **(A)** 1·10^6^ cells/scaffold, incubation with 6-OHDA in static conditions; **(B)** 0.5·10^6^ cells/scaffold, incubation with 6-OHDA under shaking (“shaken mode”). Statistical analysis was performed with two-way ANOVA for repeated measurements followed by Tukey’s multiple comparisons test. **(C)** 0.5·10^6^ cells/well, incubation with 6-OHDA in static conditions. Their metabolic activity one day after damage was considered as a control. Statistical analysis was performed with one-way ANOVA followed by Tukey’s multiple comparisons test. Ns, *p*-value > 0.05; *p-value < 0.05; ****p-value < 0.0001.

The assessment of 6-OHDA-induced neurotoxicity on SH-SY5Y cells in 3D scaffolds and its toxicity over time was run with two procedures differing from incubation mode (static/shaken).The results (Figure 4) are reported as a function of time post damage (day 0 is assumed as the day before damage in all conditions).

At first, scaffolds seeded with 1·10^6^ SH-SY5Y cells were incubated with 6-OHDA in static conditions 1 day after seeding. Oxidative damage was induced with 500 μM 6-OHDA, that reduced cell metabolic activity by 50% with respect to day 0 (*p-value < 0.05). Cells exposed to the toxic challenge recovered their metabolic activity on day 2 (ns, *p*-value > 0.05. Also on day 5 differences with day 0 were not significant, ns, *p*-value > 0.05). We also assessed a different 6-OHDA toxic concentration (250 μM), but in static conditions this incubation did not affect cell metabolic activity, that was comparable to controls at every time point examined.

Then, we performed the oxidative protocol on scaffolds seeded with 0.5·10^6^ SH-SY5Y cells, that were incubated with 6-OHDA under shaking conditions on a multifunctional rotator 4 days after seeding. The oxidative damage was induced with 6-OHDA 125 or 250 μM, that reduced cell metabolic activity by 50% and 80% with respect to day 0, respectively (****p-value < 0.0001). Three days later the damage was not recovered yet (****p-value < 0.0001).

For scaffolds seeded with 1·10^6^ SH-SY5Y cells and incubated with 500 μM 6-OHDA in static conditions, morphological analysis has suggested a reduction in cell density. However, surviving cells appeared tightly adherent to the scaffold fibers and cell clusters were still present in the most densely populated areas. For scaffolds seeded with 0.5·10^6^ SH-SY5Y cells and incubated with 125 μM 6-OHDA under shaking conditions, a change in cell morphology was detected. In fact, most cells assumed a round shape, suggestive of a stress condition.

The results for 2D controls (0.5·10^6^ SH-SY5Y cells/well, 24-well microplate) have confirmed that the selected 6-OHDA concentrations (250 or 500 μM) have a strong impact on SH-SY5Y cell metabolic activity, with a reduction of 88% and 98% in comparison to untreated controls, respectively (****p-value < 0.0001).

### Optimization of Dynamic Culture Conditions of SH-SY5Y Cells

**Figure 5:**
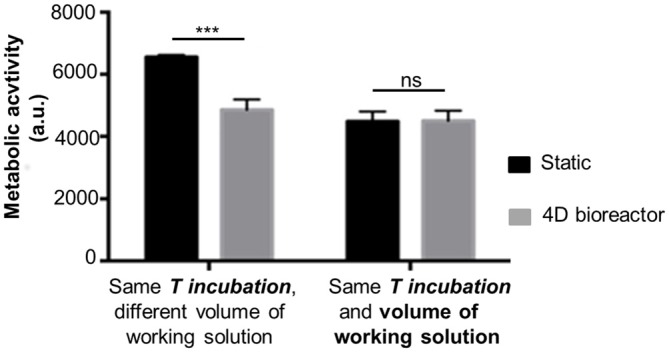
**Cell metabolic activity in 3D scaffolds: static vs. dynamic (4D bioreactor; fluorescence values, mean ± SD, *n* = 3).** Alamar Blue^®^ assay was performed on SH-SY5Y cells in 3D scaffolds in static (microplate) or dynamic (assembled in 4D bioreactor) for 3 h (T incubation). Statistical analysis was performed with one-way ANOVA followed by Tukey’s multiple comparisons test. Ns, *p*-value > 0.05; ***p-value < 0.001.

The results depicting cell metabolic activity in 3D scaffolds seeded with 0.5·10^6^ SH-SY5Y cells and kept in static or dynamic (i.e., in the 4D bioreactor) conditions are compared in Figure 5. After 3 h incubation, cell metabolic activity was greater for SH-SY5Y cells in static conditions (***p-value < 0.001). When the volume of working solution from static samples was matched to that from perfused samples by adding PBS, comparable results were obtained (ns, *p*-value > 0.05). This has led us to conclude that, as in standard static conditions, Alamar Blue^®^ assay is a reliable method to evaluate cell metabolic activity while in dynamic culture. With respect to the procedure in static conditions, the dynamic one offers the advantage of a real time monitoring of cell behavior, without needing to stop the perfusion and remove the samples from the 4D bioreactor. The initial results for static samples were greater to those from dynamic scaffolds because the lower volume of working solution (400 μl/samples instead of 900 μl) led to a greater resofurin concentration.

### Cell Recovery from the Bioreactor and Replating

After 48 h culture in the 4D bioreactor, SH-SY5Y cells were recovered from 3D scaffolds by direct extensive washing with trypsin solution. After counting, a comparable cell number was obtained for dynamic (0.46·10^6^ cells, mean value) and static samples (0.43·10^6^ cells, mean value). In addition, on day 6 the mean value of specific cell metabolic activity (that is, the ratio between cell metabolic activity, assessed by Alamar Blue^®^ assay, and cell number after detachment) was comparable between the two conditions (dynamic: 1.13 ± 0.33; static: 0.94 ± 0.19, mean ± SD).

To evaluate their capability to proliferate after dynamic culture, recovered cells were plated in a 12-well microplate. Two days later, they were collected, counted (dynamic scaffolds: 1.14·10^6^ cells; static controls: 1.28·10^6^ cells, mean values) and plated in a T25 cm^2^ flask. After further 3 days, cells (dynamic: 2.08·10^6^ cells, static controls: 4.5·10^6^ cells; mean values) were further expanded in T25 cm^2^ flasks (3 and 4 T25 cm^2^ flasks, respectively), while on day 8 after replating they were expanded in T75 cm^2^ flasks (7 and 8 T75 cm^2^ flasks for dynamic and static cells, respectively).

**Figure 6:**
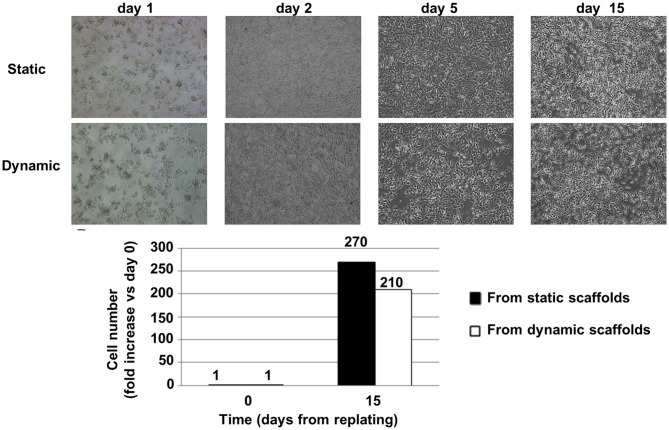
**Cell recovery from the bioreactor and replating.** Top: Brightfield images of cell morphology after replating: static vs. dynamic (48 h in the 4D bioreactor). SH-SY5Y cells were carefully detached from 3D scaffolds and plated in standard 2D conditions in a 12-well microplate (day 0 was assumed as the day of replating). On day 2 cells were moved to a T25 cm^2^ flask, on day 5 they were further expanded in T25 cm^2^ flasks, while on day 15 their serum-free conditioned culture medium was collected from T75 cm^2^ flasks for exosome isolation. Scale bar = 100 μm. Bottom: Fold increase (mean values) in cell number with respect to the day of replating. Cells were counted after detachment with trypsin.

Overall, a greater than 200-fold increase in cell number with respect to the day of replating (day 0 is assumed as the day of replating) was observed for SH-SY5Y cells recovered from dynamic and static samples. Both cell types did not show morphological alterations in comparison to standard cultured cells (Figure 6).

### Exosome Preparation from Dynamic Cultured SH-SY5Y Cells and Assessment of PRGN Exosomal Content

**Figure 7:**
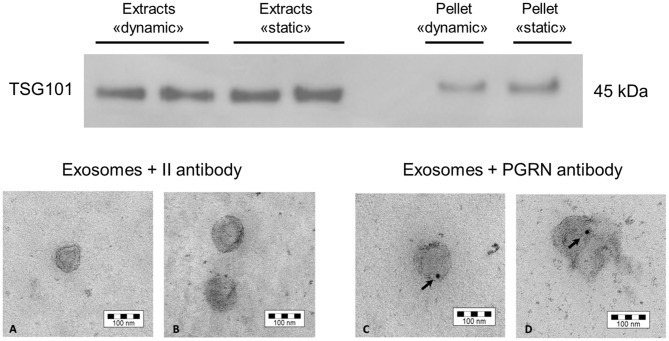
**Western blot and electron microscopy of exosomes collected from SH-SY5Y cells.** Top: Western blotting to detect the presence of the exosomal marker TSG101, both in the exosome fraction and in the whole cell protein lysate. The comparison to the static condition is also shown. Bottom: The presence of the FTLD-related protein PRGN was observed in exosomes by electron microscopy. The picture shows the control condition to exclude false positive signals due to unspecific secondary antibody reactivity **(A,B)**, while the PRGN antibody has highlighted a signal inside exosomes **(C,D)**. The reported bar is the dimension scale. PRGN: progranulin.

To test the capability of SH-SY5Y cells to secrete exosomes after exposure to shear stress due to the perfusion flow of culture medium under dynamic culture, cells were seeded in 3D scaffolds, cultured in the bioreactor for 2 days, then recovered from the scaffolds, plated and expanded as described. Exosomes were then prepared as described in “Materials and Methods” Section. We were able to efficiently isolate exosomes from cells cultured in dynamic conditions, as demonstrated by Western blotting analysis of TSG101 reactivity (Figure 7, top). A comparable ratio between the signal from cell lysate and the corresponding pellet recovered from ultracentrifugation was measured for SH-SY5Y cells kept in dynamic (0.51, mean value) or static condition (0.55, mean value), suggesting that the tested dynamic culture condition was not effective in altering exosome production phenotype of SH-SY5Y cells.

Then, we have further characterized the produced exosomes for the content of the FTLD-related protein PRGN, that has been demonstrated to be transported in these vesicles in SH-SY5Y cells as well as in fibroblasts from patients affected by genetic forms of FTLD (Benussi et al., 2016). We have performed an electron microscopy analysis on isolated exosomes to assess whether cells retain their capability to secrete PRGN in exosomes. As shown in Figure 7 (bottom), SH-SY5Y cells secrete PRGN in exosomes, even though PRGN signal was not suggestive of a strong PRGN content.

## Discussion

Microfluidic systems have attracted considerable attention in basic research as simple, reproducible and cost-efficient platforms to provide an optimal microenvironment to screen drug and cell-based therapies (Bhatia and Ingber, 2014). In accordance with this need, the miniaturized bioreactor described in the present work is optically accessible, allowing the direct and non-destructive observation of cells in 3D polystyrene scaffolds under a continuous flow of medium. The scaffolds are obtained by fuse deposition modeling, a rapid prototyping technique offering the advantage to produce highly defined, reproducible and fully interconnected architectures. Taken together, these properties allow to fully control the fluid-dynamic shear stress acting on the cells adhering to the 3D scaffolds during perfused culture, which does not exceed a shear level of 12 mPa, as predicted by computational fluid dynamic simulations (Nava et al., 2013). Thanks to the controlled perfusion of medium, the 4D bioreactor allows to maintain a stable oxygen concentration next to the cultured cells (Raimondi et al., 2015), despite the increase in cell density occurring within the cell constructs after prolonged culture.

In this work, we have optimized the 4D microfluidic bioreactor as an innovative *in vitro* culture model for the study of some FTLD-relevant features.

The optimization has begun with the seeding of neuronal-like SH-SY5Y cells in 3D scaffolds. The starting point was the procedure described for MG-63 cells (Laganà and Raimondi, 2012). MG-63 human osteoblast-like cells (ATCC^®^ CRL-1427^TM^) were seeded at a density of 1·10^6^ cells/scaffold without scaffold pre-treatment, obtaining an efficiency of 10% (that is 1·10^5^ cells were recovered 24 h later). The optimized procedure for SH-SY5Y cells has reported a greater efficiency. The adhesion of serum peptides and proteins is fundamental to promote cell adhesion (Tamada and Ikada, 1993), but polystyrene is a hydrophobic material. To overcome this limitation, 3D scaffolds were dipped overnight in culture medium. Fetal bovine serum was also tried, but cells tended to cluster and fill in the scaffold pores rather than distributing homogeneously on the fibers (data not shown). To promote cell penetration in the scaffolds while avoiding their sedimentation and aggregation, SH-SY5Y cells were seeded with a protocol alternating mixing, bending and vibration, the same applied for MG-63 cells. The use of an ultra-low attachment microplate, the placement of the scaffolds in the vertex wells and the rotation of the microplate have increased the seeding efficacy, as suggested by the reduction of the seeding time (about 3 h instead of 5 h). Optical microscopy has shown that cells are more numerous and uniform when seeded at passages between 6 and 10 (data not shown). It has also indicated that seeding 0.5·10^6^ SH-SY5Y cells/scaffold (instead of 1·10^6^) leads to a more uniform cell distribution, with few scaffold pores occluded. Overall, we have demonstrated the possibility of an efficient seeding of neuronal-like cells in the 3D scaffolds.

Then, we have focused on the development of a toxicity oxidative damage in 3D conditions also relevant for FTLD. To this purpose, we have decided to use the toxin 6-OHDA, that triggers neurotoxicity by forming free radicals and inhibiting the mitochondrial respiratory chain complexes I and IV (Glinka et al., 1997). As previously stated, mitochondrial impairment is one of the proposed pathogenic pathways involved in FTLD, and thus its modeling may be of interest. In 2D experiments, 6-OHDA is typically applied in concentrations lower than 1 mM (Storch et al., 2000). In this work two protocols were tested: the first (1·10^6^ cells incubated with 250 or 500 μM 6-OHDA) without shaking and the second (0.5·10^6^ cells incubated with 125 or 250 μM 6-OHDA) with the use of a multifunctional shaker. It is likely that the main difference between the two protocols is due to the fact that without shaking cell degeneration occurs only in the outer layers, while the use of a multifunctional rotator improves the penetration of 6-OHDA by coupling diffusion and convection. The shaking protocol is similar to that applied for cell seeding, except for the fact that in this step vibration was not set to avoid excessive cell stress. With both protocols we have observed a stable reduction of cell metabolic activity, but the second one has provided more effective and reproducible results. However, since a stable damage may be obtained with a lower toxin concentration, the treatment of 0.5·10^6^ cells with 125 μM 6-OHDA 4 days after seeding may be a preferable condition. Thus, we have been able to efficiently damage SH-SY5Y cells seeded in 3D scaffolds with a mitochondrial-targeted chemical agent. This model may represent the basis for drug screening or neuroprotective strategies in a more physiological 3D environment and dynamic context.

We have also optimized culture conditions in our 4D bioreactor to measure cell metabolic activity while in culture. It is worth to note that the assessment of the conditions in which the results from perfused scaffolds are comparable to those from similar static samples is an innovative approach. In fact, in the literature Alamar Blue^®^ or analogous assays are performed before and after cell culture in a bioreactor, not during. The main advantages of such assessment are related to the possibility to perform the assays in dynamic conditions only. In fact, the same samples can be tested at all the time points without stopping the perfusion flow of culture medium. This will allow to save samples and use the same cell culture over time as internal control, thus improving the reliability of data analysis.

A few works are available about dynamic culturing of SH-SY5Y cells. Constantinescu et al. (2007) have successfully differentiated SH-SY5Y cells to a neuronal-like state in a perfusion bioreactor. Dynamic culturing of SH-SY5Y cells has been also tried in rotary bioreactors (Morabito et al., 2015; Wang and Good, 2001). In this context, Wang and Good (2001) have observed some differences in cell viability, neurite extension and nitric oxide production between dynamic and classical static culture. In this work, we have provided original data about the effect of shear stress on SH-SY5Y cell morphology, proliferation and capability to secrete exosomes, small vesicles that are a current hotspot in investigations related to neurodegeneration and FTLD. Our results have indicated that cells retain their spindle-shaped morphology and capability to proliferate, even though cells from static scaffolds show a greater proliferation rate. Cells have also maintained their capability to secrete exosomes in culture medium and, additionally, we were able to detect in exosomes the FTLD-related protein PGRN, confirming the potential relevance of the model for this kind of dementia. To this respect, as a further refinement of our *in vitro* model, we are currently investigating the presence of PGRN in serum-free culture medium collected from the bioreactor reservoirs (outlets). As a first attempt, we have cultured SH-SY5Y cells (1·10^6^ cells/scaffold) for 72 h (48 h with serum-free medium) in the bioreactor. In these conditions, we were unable to detect the presence of exosomes in cell-conditioned medium by Western blotting, likely for an insufficient number of cells, requiring further scaling-up.

However, the development of reliable and effective *in vitro* models of neurodegeneration is strongly limited by the biological complexity of the brain, the need to produce neuronal cultures of defined cellular composition, perform quantitative measurements for validation and provide data that may be correlated with physiological outputs. Even though the assessment of functional performances in 3D systems is much more difficult than in 2D cultures (Benam et al., 2015), studies in the field of Alzheimer’s disease have highlighted that a 3D physical microenvironment and selective chemical signals are key elements to mimic tissue organization *in vivo* (Franze et al., 2013). Although biocompatible, tissue culture-treated polystyrene is stiffer than extracellular matrix and it fails to reproduce structural, chemical and physical properties of native brain tissue. To improve the mimicking of the native tissue, hydrogels have been extensively exploited (Chen et al., 2010, 2013; Bersini et al., 2014; Bang et al., 2016). Coating our 3D polystyrene scaffolds with a suitable hydrogel may improve the representativeness of our *in vitro* culture model. In particular, a collagen/poly(ethylene glycol)-based or a Carbomer/agarose-based hydrogel (Tunesi et al., 2013, 2015) that we have previously developed and characterized for central nervous system applications might be suitable candidates. The first has shown a better biological performance, but the sol-gel transition of the second is easier to be tuned in the appropriate time frame (4–15 min) for loading in the bioreactor culture chambers.

## Author Contributions

Conceived and designed the experiments: MT, MTR. Performed the experiments: MT, FeF, FaF, AC, GB. Analyzed the data: MT, FeF, FaF, MTR. Contributed reagents/materials/analysis tools: FaF. Wrote the article: MT, MTR.

## Conflict of Interest Statement

The authors declare that the research was conducted in the absence of any commercial or financial relationships that could be construed as a potential conflict of interest.

## Footnotes

^1^http://rsbweb.nih.gov/ij/
