# Supplementary material for: Optimization of a 3D Dynamic Culturing System for In Vitro Modeling of Frontotemporal Neurodegeneration-Relevant Pathologic Features
Source: Front Aging Neurosci. 2016 Jun 22;8:146. doi: 10.3389/fnagi.2016.00146 (PMC4916174; doi:10.3389/fnagi.2016.00146)
Supplement: Supplementary file 1 [file DataSheet_1.pdf]

Article title:
Optimization of a 3D dynamic culturing system for *in vitro* modeling of frontotemporal neurodegeneration-relevant pathologic features

Journal name: Frontiers in Aging Neuroscience

Author names:
Marta Tunesi, Federica Fusco, Fabio Fiordaliso, Alessandro Corbelli, Gloria Biella, Manuela T. Raimondi

Correspondence to: Marta Tunesi, Department of Chemistry, Materials and Chemical Engineering "Giulio Natta", Politecnico di Milano, P.za L. da Vinci 32, 20133, Milan, Italy.
marta.tunesi@polimi.it

Supplementary Figure 1

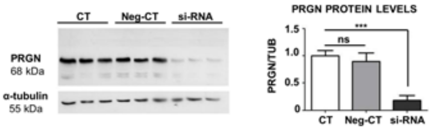

Supplementary Figure 1: Specificity of the progranulin (PRGN) antibody assessed by Western blotting. SH-SY5Y cells were treated with a small interfering RNA (siRNA) specific for PRGN and protein expression was measured. To demonstrate the specificity of the siRNA strategy, a negative control represented by a no-target siRNA (Neg-CT) was run. As internal loading control, Western blot signal was normalized to α-tubulin. The bar graph on the right is the densitometric quantification of the Western blot. Ns: not significant; ***: *p-value*<0.001, *one-way* ANOVA and post-hoc test.
